# Pan-Resistant HIV-1 Drug Resistance Among Highly Treated Patients with Virological Failure on Dolutegravir-Based Antiretroviral Therapy in Zimbabwe

**DOI:** 10.3390/v17101348

**Published:** 2025-10-08

**Authors:** Tendai Washaya, Benjamin Chimukangara, Justin Mayini, Sandra Bote, Nyasha Chin’ombe, Shungu Munyati, Justen Manasa

**Affiliations:** 1 Department of Laboratory Diagnostic and Investigative Sciences, Faculty of Medicine and Health Sciences, University of Zimbabwe, Harare P.O. Box A178, Zimbabwejmanasa@gmail.com (J.M.); 2 Biomedical Training and Research Institute, Harare P.O. Box CY 1753, Zimbabwe; jmayini@brti.co.zw (J.M.);; 3 Critical Care Medicine Department, NIH Clinical Center, Bethesda, MD 20892, USA; 4 AIDS Healthcare Foundation, Harare P.O. Box A178, Zimbabwe

**Keywords:** HIV-1, drug resistance, pan-resistance, dolutegravir

## Abstract

The HIV-1 epidemic continues to challenge global public health, especially in sub-Saharan Africa. The rise in drug-resistant viruses, particularly pan-resistant strains, threatens treatment effectiveness, hindering progress toward UNAIDS viral suppression goals. This is critical in low-to-middle income countries (LMICs) like Zimbabwe, where treatment options and access to drug resistance testing are limited. This cross-sectional study analyzed 102 genotypes from patients with HIV-1 RNA ≥ 1000 copies/mL after at least 6 months on a dolutegravir (DTG)-based ART. HIV-1 genotyping and drug resistance interpretation were performed using the Stanford HIV Drug Resistance Database. Overall, 62% of genotypes harbored at least one drug resistance mutation, with 27% showing integrase strand transfer inhibitor (INSTI)-associated mutations. High-level resistance to DTG and cabotegravir was found in 14% and 23% of integrase sequences, respectively, primarily driven by G118R and E138K/T mutations. Pan-resistance was observed in 18% of complete genotypes, with one case of four class resistance. These results highlight the emergence of INSTI resistance in LMICs. The study underscores the urgent need for enhanced HIV drug resistance testing, continuous surveillance, and strategic optimization of ART regimens in resource-constrained settings to ensure effective HIV management.

## 1. Introduction

The HIV-1 epidemic has disproportionately affected sub-Saharan Africa and continues to pose a significant public health challenge globally [1]. Despite substantial progress in HIV-1 prevention and treatment, the emergence of drug-resistant virus presents a major challenge to effective HIV-1 management [2], hindering progress towards reaching the third 95% target of the UNAIDS 95-95-95 goals, which aims for 95% of individuals on antiretroviral therapy (ART) to achieve viral suppression [3]. One particularly concerning situation is the development of pan-resistant HIV-1, where the virus acquires resistant mutations to multiple drug classes [4,5,6], including resistance to second-generation integrase strand transfer inhibitors (INSTIs) [7]. This is of particular concern in low-to-middle income countries (LMICs) where HIV-1 treatment options are limited.

Pan-resistant HIV-1 can arise from several factors, including poor adherence to ART, suboptimal therapy, and the transmission of drug-resistant strains between individuals. Lack of access to HIV drug resistance (HIVDR) testing during ART regimen switches further exacerbates the emergence and spread of pan-resistant HIV-1 in LMICs [8,9]. Like many LMICs, Zimbabwe follows a public health approach to HIV-1 treatment, starting all HIV-1 infected individuals on a first-line regimen, which for several years consisted of two nucleoside reverse transcriptase inhibitors (NRTIs) and a non-nucleoside reverse transcriptase inhibitor (NNRTI), with protease inhibitors (PIs) and INSTIs reserved for second-line and third-line regimens, respectively [10]. However, in response to the rising levels of pretreatment NNRTI-associated mutations, the country adopted the World Health Organization (WHO) recommendation to replace NNRTIs in first-line regimens with a more potent INSTI drug, i.e., dolutegravir (DTG) [10].

Currently, the first-line regimen in Zimbabwe consists of tenofovir (TDF), lamivudine (3TC), and DTG (TLD) [11]. The preferred second-line regimen for individuals failing an initial DTG-based regimen typically consists of a protease inhibitor (PI) combined with two NRTIs, without HIV genotypic testing. Patients previously unable to achieve viral suppression on an EFV-based first-line regimen are switched to a DTG-based second-line regimen without HIV genotypic testing. This is due to the limited resources available for HIVDR testing in Zimbabwe, which is primarily reserved for patients failing second-line therapy before switching to third-line regimens. As a result, these patients may develop resistance to three classes of antiretroviral drugs before receiving an HIV genotypic drug resistance test to determine viral susceptibility.

Although clinical trials have shown that DTG retains some efficacy against HIVDR strains, including those with INSTI mutations [12,13], virological failure has been reported in individuals receiving DTG, often due to poor adherence and inadequate treatment monitoring [14,15,16]. Therefore, this study sought to assess pan-resistant HIV-1 among DTG-treated individuals in Zimbabwe and evaluate the extent of reduced ART efficacy, providing insights to guide public health decisions in HIV-1 treatment management.

## 2. Materials and Methods

### 2.1. Study Design

This was a cross-sectional study aimed at assessing pan-resistant HIV-1 among highly treatment-experienced patients with virological failure on DTG-based second-line ART in Zimbabwe. Virological failure was defined as having at least two consecutive HIV-1 viral loads (VLs) ≥ 1000 copies/mL of plasma after at least six months on DTG-based ART. Plasma samples from patients meeting these criteria were obtained during the period January 2021 to July 2024, through the Zimbabwe National HIVDR Testing Program. This is a public health initiative designed to expand access to HIVDR testing across public healthcare facilities in Zimbabwe, allowing for early detection of individuals with HIVDR, which is essential for optimizing treatment strategies and preventing the spread of drug-resistant virus within communities. The samples obtained in this study were collected from all provinces of Zimbabwe, through a national referral network. However, most samples originated from three major provinces, namely Harare, Bulawayo, and Mashonaland West provinces. Demographic and clinical information were obtained from patient request forms.

### 2.2. Laboratory Methods

HIV-1 VL testing was performed at the respective hospitals providing HIV-1 care. Whole blood samples from patients with virological failure (i.e., VLs ≥ 1000 copies/mL) were sent for HIV-1 genotyping at the Biomedical Research and Training Institute (BRTI) in Harare. Viral HIV-1 RNA was extracted from plasma samples using the NucliSens easyMAG system (bioMérieux) and amplified using the Applied Biosystems TaqPath Seq HIV-1 Genotyping Assay Kit (ThermoFisher Scientific, Waltham, MA, USA), according to manufacturers’ instructions. The quality of the nested PCR product was assessed on a 1% agarose gel against an O’GeneRuler 1kb DNA ladder as a molecular weight marker. Successfully amplified samples were sequenced for the protease (PR), reverse transcriptase (RT), and integrase (IN) genes, on a SeqStudio Genetic Analyzer (ThermoFisher Scientific, Waltham, MA, USA).

Sequence data were analyzed using Geneious Prime software (2024.0.7) [17] which assigns a quality score to each sequence; scores above 70% were considered indicative of high-quality sequences. HIVDR mutations and viral subtypes were determined using the Stanford HIV Drug Resistance Database, version 9.8 [18]. HIVDR was defined as having at least one mutation causing potential-low level resistance (PLLR) to high-level resistance (HLR) to any PI, NRTI, NNRTI, or INSTI drug used in the standard of care for HIV-1 treatment in Zimbabwe. We assigned provincial codes to each sequence based on its province of origin. Maximum likelihood phylogenetic tree reconstruction was performed to assess geographical clustering using a generalized time reversible model, with proportion of invariable sites and gamma distribution (GTR + I + G) in the Geneious Prime software (2024.0.7) [17]. Bootstrap replicates (100) were applied for internal node support. Sequence data have been deposited in GenBank under accession numbers PX097068–PX097169.

### 2.3. Statistical Analysis

Logistic regression and chi-square tests were used to assess associations between HIVDR and patient characteristics, namely age, sex, VL, current ART regimen, and duration on ART. Descriptive statistics were used to analyze HIVDR mutations, and all statistical analyses were performed using Stata software, version 17.0 (StataCorp LP, College Station, TX, USA; 800-STATA-PC).

## 3. Results

**Figure 1:**
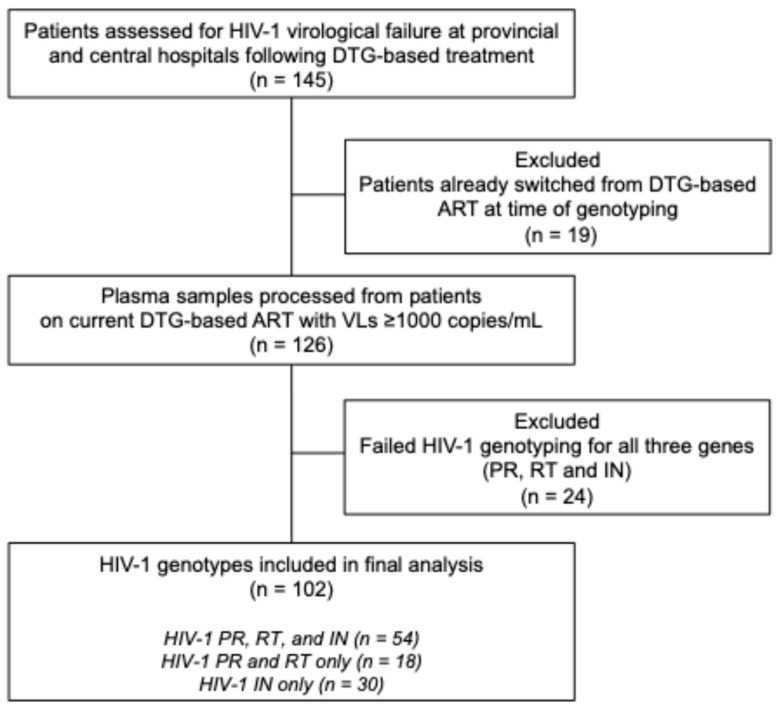
Samples processed for HIV-1 genotyping from patients failing DTG-based treatment in Zimbabwe. ART, antiretroviral therapy; DTG, dolutegravir; IN, *integrase* gene; mL, milliliter; PR, *protease* gene; RT, *reverse transcriptase* gene; VLs, viral loads.

Of 145 plasma samples obtained from patients with virological failure following DTG-based treatment, 19 (13.1%) had switched from a DTG-based regimen, and 24 (16.6%) failed genotyping, and were therefore excluded from further analysis. Of 102 analyzed, 54 (52.9%) had complete genotypes for PR, RT, and IN genes, while the remaining had either complete PR and RT genotypes (18, 17.7%), or IN genotypes only (30, 29.4%) (Figure 1).

Most patients (75, 73.5%) were between the ages of 15–49, and there were more females than males (55.9% vs. 44.1%) (Table 1). Approximately four in every nine patients were receiving a regimen containing tenofovir disoproxil fumarate (TDF), lamivudine (3TC), and DTG at the time of HIV-1 genotyping (Table 1). The majority of patients (73, 71.6%) had prior exposure to NNRTIs (i.e., efavirenz (EFV) or nevirapine (NVP)), approximately one third had been exposed to zidovudine (AZT) (32, 31.4%), whilst some had prior exposure to PIs (29, 28.4%), namely ritonavir-boosted atazanavir (ATZ/r) and ritonavir-boosted lopinavir (LPV/r).

### 3.1. HIV-1 Drug Resistance Mutations

Of the 102 HIV-1 genotypes included in the final analysis, 63 (61.8%) had at least one HIVDR mutation detected. Patients with HIVDR (24 years, 95% confidence interval (CI): 21–29) were relatively younger compared to those without HIVDR (27 years, 95% CI: 21–37) (Table 2), and males had a higher proportion of HIVDR (31/45, 68.9%) compared to females (32/57, 56.1%), *p* = 0.222 (Fisher’s exact test). Most patients (50, 57.5%) had received DTG-based ART for over 24 months, with those harboring HIVDR having a longer treatment duration compared to those without HIVDR. Most patient samples were obtained from three of ten provinces (i.e., Bulawayo, Harare, and Mashonaland West), where the country’s main central hospitals are located (Table 2).

Of the 102 sequences, 72 (70.6%) had a PR and RT genotype, and 84 (82.4%) had an IN genotype. Of the 72 with a PR and RT genotype, 51 (70.8%) had at least one HIVDR mutation. Of those, 11 (15.3%) had PI resistance, and 36 (50.0%) had NRTI resistance. The most common major PI mutations were M46ILV, V82AL, and N88S, all occurring in 4/72 (5.6%) genotypes. The NRTI mutation M184V was the most common overall, occurring in 29/72 (40.3%) genotypes, while 26 (36.1%) genotypes harbored thymidine analog mutations (TAMs), 10 (13.9%) of which had no known prior exposure to thymidine analogs (i.e., atypical TAMs) (Supplementary Table S1).

**Figure 2:**
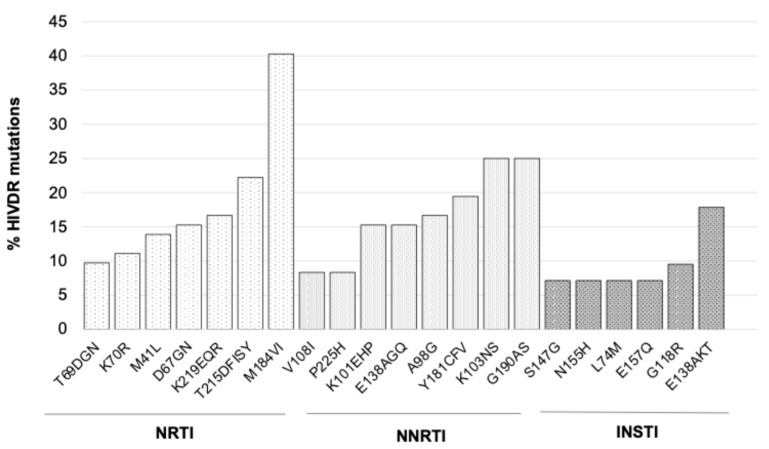
Most common HIV-1 drug resistance mutations detected in treatment-experienced patients with virological failure on DTG-based ART in Zimbabwe. HIVDR, HIV drug resistance; INSTI, integrase strand transfer inhibitor; NNRTI, non-nucleoside reverse transcriptase inhibitor; NRTI, nucleoside reverse transcriptase inhibitor. Only mutations observed at >7% are shown in this figure.

Despite all patients receiving non-NNRTI-based ART at the time of virological failure, approximately three in every five RT genotypes (45, 62.5%) had NNRTI resistance mutations, with K103NS (18, 25.0%) and G190AS (18, 25.0%) being the most common NNRTI mutations detected (Figure 2). Among the 84 IN genotypes, 23 (27.4%) had at least one INSTI mutation, 3 (3.6%) of which had INSTI-accessory mutations only, namely H51Y and E157Q (Supplementary Table S2). E138AKT was the most common INSTI-major mutation detected (15, 17.9%), whilst approximately one in every ten genotypes (8, 9.5%) harbored the G118R mutation (Figure 2 and Supplementary Table S3). The IN mutations E138AKT and G118R cause PLLR and HLR to DTG, respectively. Figure 2 summarizes the most frequently detected mutations, and Supplementary Table S2 shows details of all HIVDR mutations detected.

We further assessed the levels of resistance to individual antiretroviral drugs and found that most genotypes harbored viruses susceptible to PIs. However, approximately one in eight genotypes exhibited HLR to atazanavir (ATV) (Table 3). Among the NRTIs, the majority of genotypes remained susceptible to tenofovir (TDF), with fewer than 5% showing HLR. In contrast, nearly one in four genotypes displayed intermediate to HLR to abacavir (ABC). About two-thirds of genotypes had either PLLR or remained fully susceptible to etravirine (ETR). Although susceptibility patterns were similar between etravirine and rilpivirine (RPV), a higher proportion of genotypes exhibited HLR to RPV (Table 3). Genotypes with integrase mutations typically showed HLR to INSTIs, primarily driven by G118R and E138AKT mutations (Table 3 and Figure 2). Notably, cabotegravir (CAB) was associated with the highest resistance levels among INSTIs, while DTG and bictegravir (BIC) demonstrated comparable resistance profiles (Table 3, Figure 2, and Supplementary Table S4).

### 3.2. Drug Class Resistance and Phylogenetic Clustering

**Figure 3:**
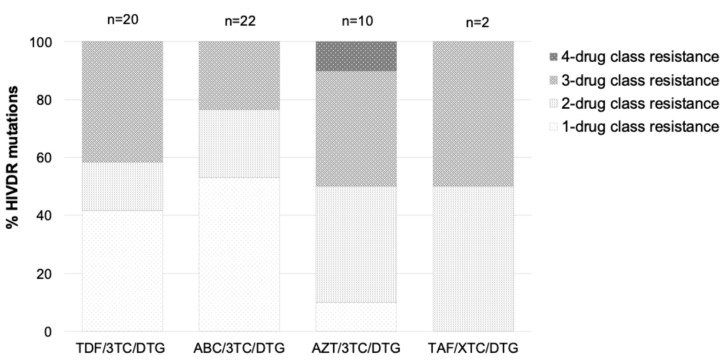
Characterization of antiretroviral drug resistance in 54 HIV-1 genotypes from patients failing DTG-based regimens in Zimbabwe. 3TC, lamivudine; ABC, abacavir; AZT, zidovudine; DTG, dolutegravir; HIVDR, HIV drug resistance; TAF, tenofovir alafenamide; TDF, tenofovir disoproxil fumarate; XTC, lamivudine or emtricitabine.

We further assessed the extent of drug class resistance among the 54 genotypes with all three genes, i.e., PR, RT, and IN. Of the 54, 13 (24.1%) had single-class resistance, mainly NNRTI-associated resistance (8/15, 53.3%). Dual-class resistance was observed in 11 (20.4%) genotypes, 8 (14.8%) with NRTI and NNRTI resistance, whilst 15 (27.9%) had triple-class resistance, and 1 (1.9%) had resistance to all four drug classes. The proportions of HIV-1 drug resistance by drug regimen, among the 54 genotypes, are shown in Figure 3 and Supplementary Table S5.

**Figure 4:**
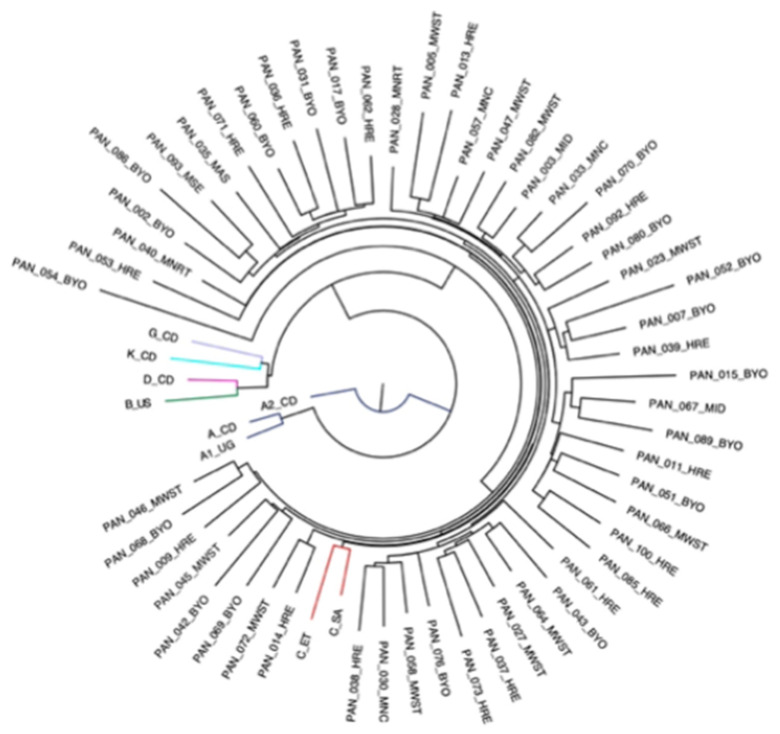
Maximum likelihood phylogenetic tree showing clustering of HIV sequences from patients assessed for drug resistance in PR, RT, and IN genes in Zimbabwe. Each tip represents an individual patient sequence and subtype references used are color-coded: subtype A (blue), subtype B (green), subtype C (red), subtype D (pink), subtype K (turquoise), and subtype G (purple). The tree was constructed using a generalized time reversible model, with a proportion of invariable sites and gamma distribution (GTR+I+G), with 100 bootstrap replicates for internal node support. BYO, Bulawayo; HRE, Harare; MNRT, Manicaland; MWST, Mashonaland West; MSE, Mashonaland East; MAS, Masvingo; MNC, Matabeleland North; MID, Midlands.

Phylogenetic analysis was performed on the 54 genotypes with complete PR, RT, and IN genotypes. All sequences clustered around HIV-1 subtype C, and there was no geographical clustering observed across all provinces (Figure 4). Reference sequences included in phylogenetic analyses have colored external branches, and patient sequences generated from this study have assigned provincial codes.

## 4. Discussion

In this study, which assessed pan-resistance among treatment-experienced patients with virological failure on DTG-based ART in Zimbabwe, we found that approximately 27% of genotypes harbored INSTI-associated drug-resistant mutations, with nearly 15% exhibiting HLR to DTG. These findings are consistent with results from the DTG RESIST study, evaluating DTG resistance in non-B HIV subtypes in seven African countries, which reported 26% INSTI resistance, including 21% with HLR to DTG [19]. Interestingly, a real-world cohort study from Mozambique reported even higher DTG resistance levels (46%), though based on a relatively small sample size [20].

While over 60% of patients in our study had at least one HIVDR mutation, a smaller subset (17.7%) exhibited pan-resistance, i.e., resistance to three or more drug classes, with only one patient having HIVDR to all four major antiretroviral drug classes, namely PIs, NRTIs, NNRTIs, and INSTIs. These findings suggest that, although INSTI resistance may be more common in non-clinical trial settings, complete pan-resistance remains relatively uncommon, even among those with long-term ART exposure. Additionally, we observed that individuals with HIVDR mutations had been on DTG-based regimens for longer durations than those without resistance, supporting existing evidence that prolonged treatment is a key driver of resistance accumulation [21].

With the growing use of INSTIs in both HIV treatment and prevention, a key concern emerging from this study is the relatively high proportion (~23%) of patients whose virus harbors HLR to CAB, a key drug approved by the World Health Organization (WHO) in recent years as a long-acting injectable (CAB-LA) for pre-exposure prophylaxis (PrEP) [22]. Notably, about one in four genotypes also showed HLR to rilpivirine, which is commonly co-administered with CAB. These findings raise important concerns regarding the efficacy of CAB-LA for PrEP, and have significant implications for its broader use in both treatment and prevention contexts [23], underscoring the need for careful surveillance of individuals currently using this regimen. The most frequently observed mutations associated with INSTI resistance were G118R and E138KT, mirroring findings from other studies involving non-B HIV subtypes, including the DTG RESIST study [19]. Both mutations confer cross-resistance to multiple INSTIs, with G118R, in particular, associated with higher levels of INSTI resistance [1]. These patterns highlight serious concerns about the long-term effectiveness of INSTIs, especially in settings where non-B subtypes are predominant.

Importantly, most genotypes in our study retained full or partial susceptibility to TDF, with only five cases (6.9%) carrying the K65R mutation and fewer than 5% exhibiting HLR to the drug. These findings support the continued use of TDF as a dependable NRTI in both first- and second-line ART, and potentially in people experiencing failure on INSTI-based regimens. Notably, the most frequently observed NRTI mutations were TAMs, occurring at RT positions 41, 67, 70, 215, and 219 (Figure 2), which primarily confer resistance to thymidine analogs like AZT [18]. Of note, approximately 13% of patients had atypical TAMs, highlighting the importance of genotypic resistance testing in treatment-experienced individuals prior to regimen switches. A cross-sectional study from a hyper-endemic HIV region in South Africa reported even higher rates (>40%) of atypical TAMs among patients on TDF-based ART, underscoring the need for further research into the emergence and mechanisms of TAMs in the context of non-thymidine analogue use [24]. Despite the presence of TAMs, our findings suggest that AZT may be a preferred NRTI alternative to ABC, with approximately 67% of genotypes maintaining susceptibility to AZT compared to 57% for ABC. Susceptibility to ABC was frequently compromised by the M184V mutation. These considerations are particularly critical in LMICs, where treatment options are limited and access to routine genotypic resistance testing remains a challenge.

Although none of the patients were receiving NNRTI-based therapy at the time of virologic failure, NNRTI resistance mutations were the most frequently observed, present in approximately 63% of genotypes, most commonly K103NS and G190AS. Persistence of NNRTI resistance has previously been documented in second-line failures, with NNRTI resistance mutations detected in ~90% of genotypes, even though only 1% of patients were receiving NNRTI-containing regimens at the time of genotyping [25]. Interestingly, around two-thirds of genotypes remained either fully susceptible or demonstrated PLLR to ETR, a second-generation NNRTI known for its higher resistance barrier. This suggests that ETR could be a viable option in future regimens for heavily treated patients [26], particularly in settings where PI-based options are limited or contraindicated.

Among all drug classes assessed, PIs exhibited the lowest levels of resistance, with only 15% of genotypes carrying PI-associated mutations. Most genotypes remained fully susceptible to lopinavir (LPV), ATV, and darunavir (DRV). Resistance was most pronounced with ATV, while DRV showed the highest robustness, with only 6% of genotypes exhibiting any resistance (Table 3). These findings reinforce the role of PIs as a strong alternative for patients experiencing failure due to resistance to other drug classes, especially when INSTI-based regimens are compromised. Despite the PI drugs appearing largely effective, the high level of resistance to atazanavir (12.5% of genotypes) may limit its clinical utility in a subset of patients. This underscores the need to evaluate PI resistance at patient-level, rather than generalizing across the entire population. Lastly, no clustering of sequences by province of sample origin was observed, indicating a uniform pattern of ART regimen use across the country, consistent with national standard-of-care guidelines.

These findings should be interpreted in light of the following limitations. First, treatment adherence, an important factor contributing to both virologic failure and the development of drug resistance, was not assessed, due to the nature of samples obtained. As such, our findings should be interpreted primarily to describe resistance profiles present in INSTI-treated patients with virological failure, rather than a definitive explanation of resistance mechanisms. Second, although samples were collected from multiple provinces and healthcare facilities across Zimbabwe, the majority originated from Harare, Bulawayo, and Mashonaland West provinces. Notably, some provinces were underrepresented or not represented at all, which limits the generalizability of our findings to the entire country. Future surveillance efforts should aim for more balanced geographical representation to provide a comprehensive national profile of HIVDR, particularly as access to DTG and other INSTIs expands across all levels of the healthcare system.

Third, we excluded 43 individuals, 19 of whom had transitioned from DTG-based regimens and 24 of whom had unsuccessful genotyping. Thus, the number of samples processed may not fully reflect the complexity of resistance dynamics in the broader population of individuals experiencing virological failure. However, our study focused only on people currently receiving DTG-based ART, as resistance profiles are expected to shift under ongoing drug pressure. Moreover, genotyping of samples with lower viral loads is often challenging, warranting future studies addressing these limitations. Lastly, complete genotyping data for all three relevant genes (i.e., PR, RT, IN) were available for only a subset of samples, reducing the number of genotypes included in the analysis of multi-drug resistance. However, despite these limitations, this study is the first in Zimbabwe to examine pan-resistance in the context of INSTI-based ART regimens within public healthcare settings, providing crucial data to inform future treatment strategies.

In conclusion, this study provides valuable real-world insights into the evolving landscape of HIVDR among individuals experiencing virological failure on DTG-based ART in a resource-constrained setting. While pan-resistance remains infrequent, the emergence of key INSTI mutations, particularly those conferring HLR to long-acting CAB, is a cause for clinical and public health concern. Strategic use of AZT, ETR, and PIs may play a pivotal role in maintaining regimen potency and ensuring the durability of ART in similar high-burden, low-resource contexts. Overall, these findings underscore the critical importance of sustained molecular surveillance, equitable expansion of HIVDR testing, and evidence-based regimen optimization.

## Figures and Tables

**Table 1:** Demographics and clinical characteristics of patients assessed for pan-resistant HIV-1 in Zimbabwe.

Variable	*n*, 102	%
**Age groups ^a^**		
<15	12	11.8
15–24	37	36.3
25–49	38	37.3
>50	10	9.8
**Sex**		
Male	45	44.1
Female	57	55.9
Months since ART initiation, median (IQR) ^b^	139 (107–180)	-
pVL log_10_ (copies/mL), median (IQR)	4.7 (4.1–5.3)	-
**ART regimen at enrollment**		
TDF/3TC/DTG	47	46.1
ABC/3TC/DTG	34	33.3
AZT/3TC/DTG	18	17.7
TAF/XTC/DTG	3	2.9

3TC, lamivudine; ABC, abacavir; ART, antiretroviral therapy; AZT, zidovudine; DTG, dolutegravir; IQR, interquartile range; mL, milliliter; pVL, plasma viral load, TAF, tenofovir alafenamide; TDF, tenofovir disoproxil fumarate; XTC, lamivudine or emtricitabine. ^a^ Five patients had missing data for age. ^b^ Eighteen patients had missing data for months on ART.

**Table 2:** Characteristics of patients with and without HIV-1 drug resistance assessed for pan-resistance in Zimbabwe.

Characteristics	All HIV-1 Genotypes (*n* = 102)	Genotypes with HIVDR (*n* = 63)	Genotypes Without HIVDR (*n* = 39)
Age in years, (95% CI) ^a^	24 (22–30)	24 (21–29)	27 (21–37)
**Sex**			
Males, *n* (%)	45 (44.1)	31 (49.2)	14 (35.9)
Females, *n* (%)	57 (55.8)	32 (50.8)	25 (64.1)
Months on DTG-based ART, median (IQR) ^b^	24 (17–36)	24 (12–36)	25 (18–36)
Months on ART, median (IQR) ^c^	139 (107–180)	141 (109–184)	118 (86–175)
**Current ART regimen, (%)**			
TDF/3TC/DTG	47 (46.1)	24 (38.1)	23 (59.0)
ABC/3TC/DTG	34 (33.3)	24 (38.1)	10 (25.6)
AZT/3TC/DTG	18 (17.7)	13 (20.6)	5 (12.8)
TAF/XTC/DTG	3 (2.9)	2 (3.2)	1 (2.6)
pVL log_10_ copies/mL, median (IQR) ^d^	4.7 (4.1–5.3)	4.7 (4.2–5.3)	4.8 (4.0–5.3)
**Province ^e^**			
Bulawayo	35 (34.3)	21 (33.3)	14 (35.9)
Harare	26 (25.5)	18 (28.6)	8 (20.5)
Masvingo	2 (2.0)	1 (1.6)	1 (2.6)
Midlands	3 (2.9)	3 (4.8)	0 (0)
Manicaland	4 (3.9)	2 (3.2)	2 (5.1)
Matabeleland North	7 (6.9)	4 (6.4)	3 (7.7)
Mashonaland East	1 (1.0)	1 (1.6)	0 (0)
Mashonaland West	24 (23.5)	13 (20.6)	11 (28.2)

3TC, lamivudine; ABC, abacavir; ART, antiretroviral therapy; AZT, zidovudine; CI, confidence interval; DTG, dolutegravir; HIVDR, HIV drug resistance; IQR, interquartile range; mL, milliliter; pVL, plasma viral load; TAF, tenofovir alafenamide; TDF, tenofovir disoproxil fumarate; XTC, lamivudine or emtricitabine. ^a^ Five patients had missing data for age. ^b^ Fifteen patients had missing data for months on DTG-based ART. ^c^ Eighteen patients had missing data for months on ART. ^d^ Five patients had missing data for viral loads. ^e^ There were no samples obtained from 2 of the 10 provinces in Zimbabwe.

**Table 3:** Levels of HIVDR conferred to individual antiretroviral drugs among patients assessed for pan-resistant HIV in Zimbabwe.

Antiretroviral Drug	Level of Resistance				No Resistance
	High	Intermediate	Low	Potential-Low	
**PI (n = 72)**					
Lopinavir	2 (2.8)	4 (5.6)	0 (0)	3 (4.2)	63 (87.5)
Atazanavir	9 (12.5)	0 (0)	2 (2.8)	0 (0)	61 (84.7)
Darunavir	1 (1.4)	0 (0)	3 (4.2)	0 (0)	68 (94.4)
**NRTI (n = 72)**					
Tenofovir	3 (4.2)	9 (12.5)	9 (12.5)	2 (2.8)	49 (68.1)
Lamivudine	29 (40.3)	0 (0)	1 (1.4)	0 (0)	42 (58.3)
Abacavir	15 (20.8)	9 (12.5)	7 (9.7)	0 (0)	41 (56.9)
Zidovudine	15 (20.8)	4 (5.6)	4 (5.6)	1 (1.4)	48 (66.7)
**NNRTI (n = 72)**					
Efavirenz	34 (47.2)	8 (11.1)	1 (1.4)	2 (2.78)	27 (37.5)
Nevirapine	43 (59.7)	1 (1.4)	0 (0)	1 (1.4)	27 (37.5)
Etravirine	10 (13.9)	11 (15.3)	5 (6.9)	12 (16.7)	34 (47.2)
Rilpivirine	18 (25)	9 (12.5)	11 (15.3)	1 (1.4)	33 (45.8)
**INSTI (n = 84)**					
Dolutegravir	12 (14.3)	7 (8.3)	0 (0)	3 (3.6)	62 (73.8)
Bictegravir	11 (13.1)	8 (9.5)	0 (0)	3 (3.6)	62 (73.8)
Cabotegravir	19 (22.6)	0 (0)	3 (3.6)	0 (0)	62 (73.8)
Elvitegravir	16 (19.1)	3 (3.6)	3 (3.6)	1 (1.2)	61 (72.6)
Raltegravir	16 (19.1)	1 (1.2)	5 (6.0)	1 (1.2)	61 (72.6)

INSTI, integrase strand transfer inhibitor; NNRTI, non-nucleoside reverse transcriptase inhibitor; NRTI, nucleoside reverse transcriptase inhibitor; PI, protease inhibitor.

## Data Availability

The original contributions presented in this study are included in the article/Supplementary Material. Further inquiries can be directed to the corresponding author.

## Supplementary Materials

The following supporting information can be downloaded at https://www.mdpi.com/article/10.3390/v17101348/s1. Table S1: Levels of resistance to NRTI drugs among patients with atypical thymidine analog mutations (TAMs) in Zimbabwe; Table S2: HIV-1 drug resistance mutations detected in genotypic resistance tests from patients failing DTG-based regimens in Zimbabwe; Table S3: Levels of resistance to INSTI drugs among patients habouring INSTI-associated mutations in Zimbabwe; Table S4: Susceptibility of individual antiretroviral drugs among patients assessed for pan-resistant HIV in Zimbabwe; Table S5: Drug class HIV-1 resistance patterns observed in patients failing DTG-based ART in Zimbabwe.

## Abbreviations

The following abbreviations are used in this manuscript:

HIV	Human Immunodeficiency Virus
ART	Anti-retroviral therapy
LMIC	Low- and middle-income countries
HIVDR	Human Immunodeficiency virus drug resistance
NRTI	Nucleotide reverse transcriptase
NNRTI	Non-nucleotide reverse transcriptase
PI	Protease inhibitors
INSTI	Integrase strand transfer inhibitors
PR	Protease
RT	Reverse transcriptase
IN	Integrase
WHO	World Health Organization
TLD	Tenofovir Lamivudine Dolutegravir
TDF	Tenofovir
EFV	Efavirenz
DTG	Dolutegravir
3TC	Lamivudine
NVP	Nevirapine
AZT	Zidovudine
LPV/r	Lopinavir boosted with ritonavir
ETR	Etravirine
RPV	Rilprinavir
PLLR	Potential low-level resistance
HLR	High level resistance
VL	Viral load
