# Supplementary material for: Pan-Resistant HIV-1 Drug Resistance Among Highly Treated Patients with Virological Failure on Dolutegravir-Based Antiretroviral Therapy in Zimbabwe
Source: Viruses. 2025 Oct 8;17(10):1348. doi: 10.3390/v17101348 (PMC12567580; doi:10.3390/v17101348)
Supplement: Supplementary file 1 [file viruses-17-01348-s001.zip › viruses-3776771-supplementary.pdf]

SUPPLEMENTARY TABLES

Supplementary Table S1 Levels of resistance to NRTI drugs among patients with atypical thymidine analogue mutations (TAMs) in Zimbabwe.

Study ID	NRTI-backbone	NRTI mutations	Levels of resistance						
			**ABC**	**AZT**	**D4T**	**DDI**	**FTC**	**3TC**	**TDF**
PAN_005	ABC	**M41L**, S68G, V75T, M184V, **L210W**, **T215Y**	HLR	HLR	HLR	HLR	HLR	HLR	IR
PAN_007	ABC	**D67N**, T69D	S	LLR	LLR	IR	S	S	S
PAN_011	ABC	**M41L**	S	LLR	LLR	PLLR	S	S	S
PAN_023	ABC	**K70R**, M184V, **K219Q**	LLR	IR	LLR	IR	HLR	HLR	S
PAN_036	ABC	D67G, S68G, **K70R**, L74I, M184V, **K219E**	HLR	HLR	HLR	HLR	HLR	HLR	IR
PAN_047	ABC	K65R, S68G, M184V, **K219R**	HLR	S	HLR	HLR	HLR	HLR	IR
PAN_052	TDF	**M41L**, E44D, T69D, V75M, **L210W**, **T215Y**, **K219R**	HLR	HLR	HLR	HLR	LLR	LLR	HLR
PAN_053	TDF	**D67N**, **K70R**, M184V, **K219Q**	IR	HLR	IR	IR	HLR	HLR	LLR
PAN_083	ABC	**M41L**, **D67N**, L74I, M184V, **T215Y**	HLR	HLR	HLR	HLR	HLR	HLR	IR
PAN_093	ABC	**M41L**, V75I, M184V, **T215Y**	IR	HLR	HLR	IR	HLR	HLR	LLR

S, susceptible; PLLR, potential low-level resistance; LLR low-level resistance; IR, intermediate resistance; HLR, high-level resistance; ABC, abacavir; AZT, zidovudine; D4T, stavudine; DDI, didanosine; FTC, emtricitabine, TDF, tenofovir

All patient regimens included 3TC and DTG. Atypical TAMs (**bold**) are classified as thymidine analogue mutations occurring in patients with no known prior exposure to AZT or D4T.

Supplementary Table S2 HIV-1 drug resistance mutations detected in genotypic resistance tests from patients failing DTG-based regimens in Zimbabwe.

Mutation	Frequency	Proportion (%)
**PI mutations (n=72)**		
V32I	1	1.4
M46ILV	4	5.6
I50L	2	2.8
I54LV	2	2.8
L76V	1	1.4
V82AL	4	5.6
I84V	4	5.6
N88S	4	5.6
L90M	3	4.2
L10F	4	5.6
K20T	4	5.6
L24IV	1	1.4
L33F	2	2.8
K43T	1	1.4
Q58E	3	4.2
G73ST	4	5.6
**NRTI Mutations (n=72)**		
M41L	10	13.9
K65RN	5	6.9
D67GN	11	15.3
T69DGN	7	9.7
K70ET	2	2.8
K70R	8	11.1
L74FIV	5	6.9
V75IMT	5	6.9
F77L	1	1.4
Y115F	4	5.6
M184VI	29	40.3
L210W	2	2.8
T215DFISY	16	22.2
K219EQR	12	16.7
**NNRTI mutations (n=72)**		
A98G	12	16.7
L100I	2	2.8
K101EHP	11	15.3
K103NS	18	25.0
V106AILM	5	6.9
V108I	6	8.3
E138AGQ	11	15.3

V179DE	3	4.2
Y181CFV	14	19.4
Y188CFL	2	2.8
G190AS	18	25.0
H221Y	2	2.8
P225H	6	8.3
F227LV	1	1.4
**INSTI mutations (n=84)**		
T66AI	4	4.8
G118R	8	9.5
E138AKT	15	17.9
G140A	4	4.8
S147G	6	7.1
Q148KR	4	4.8
N155H	6	7.1
R263K	4	4.8
H51Y	2	2.4
L74M	6	7.1
Q95K	1	1.2
T97A	4	4.8
E157Q	6	7.1
G163RK	2	2.4

GRT, genotypic resistance test; INSTI, integrase strand transfer inhibitor; NNRTI, non-nucleoside reverse transcriptase inhibitor; NRTI, nucleoside reverse transcriptase inhibitor; PI, protease inhibitor.

Supplementary Table S3 Levels of resistance to INSTI drugs among patients habouring INSTI-associated mutations in Zimbabwe.

	Study ID	INSTI mutations	Levels of resistance				
			BIC	CAB	DTG	EVG	RAL
1	PAN_005	E138K, G140A, S147G, Q148R, N155H	HLR	HLR	HLR	HLR	HLR
2	PAN_006	*T97A*, S147G, N155H	IR	HLR	IR	HLR	HLR
3	PAN_013	*L74M*, G118R, E138K, R263K	HLR	HLR	HLR	HLR	HLR
4	PAN_017	*Q95K*, E138A, G140A, S147G, Q148R, N155H	HLR	HLR	HLR	HLR	HLR
5	PAN_021	*L74M*, G118R, E138K	HLR	HLR	HLR	HLR	HLR
6	PAN_024	G118R, E138K	HLR	HLR	HLR	HLR	HLR
7	PAN_041	*L74M*, E138K	PLLR	LLR	PLLR	LLR	LLR
8	PAN_042	R263K	IR	HLR	IR	IR	LLR
9	PAN_043	E138K, G140A, Q148K, *G163R*	HLR	HLR	HLR	HLR	HLR
10	PAN_046	*E157Q*	S	S	S	PLLR	PLLR
11	PAN_047	R263K	IR	HLR	IR	IR	LLR
12	PAN_049	T66I, G118R, E138K	HLR	HLR	HLR	HLR	HLR
13	PAN_053	*T97A*, E138K, S147G, N155H	IR	HLR	IR	HLR	HLR
14	PAN_054	*H51Y*	PLLR	LLR	PLLR	LLR	LLR
15	PAN_073	E138K, G140A, Q148R, *E157Q*	HLR	HLR	HLR	HLR	HLR
16	PAN_077	*T97A*, E138K, S147G, N155H	IR	HLR	IR	HLR	HLR
17	PAN_079	T66A, L74M, G118R, E138AT	HLR	HLR	HLR	HLR	HLR
18	PAN_090	*T97A*, E138K, S147G,N155H	IR	HLR	IR	HLR	HLR
19	PAN_092	*H51Y*, *E157Q*	PLLR	LLR	PLLR	LLR	LLR
20	PAN_094	*E157Q*, R263K	IR	HLR	IR	IR	IR
21	PAN_097	T66A, G118R, E138K, *E157Q*	HLR	HLR	HLR	HLR	HLR
22	PAN_101	T66A, *L74M*, G118R, E138A, *E157Q*	HLR	HLR	HLR	HLR	HLR
23	PAN_102	*L74M*, G118R, *G163K*	IR	HLR	HLR	HLR	HLR

S, susceptible; PLLR, potential low-level resistance; LLR low-level resistance; IR, intermediate resistance; HLR, high-level resistance; BIC, bictegravir; CAB, cabotegravir; DTG, dolutegravir; EVG, elvitegravir; RAL, raltegravir

Italics represent INSTI-accessory mutations

Supplementary Table S4 Susceptibility of individual antiretroviral drugs among patients assessed for pan-resistant HIV in Zimbabwe.

ARV drugs	Level of resistance				No resistance
	High	Intermediate	Low	Potential-low	
**PI (n = 72)**					
Lopinavir	2 (2.8)	4 (5.6)		3 (4.2)	63 (87.5)
Nelfinavir	*9 (12.5)*	*2 (2.8)*	*1 (1.4)*	*1 (1.4)*	*59 (81.9)*
Atazanavir	9 (12.5)		2 (2.8)		61 (84.7)
Darunavir	1 (1.4)		3 (4.2)		68 (94.4)
Fosamprenavir	*5 (6.9)*	*1 (1.4)*	*3 (4.2)*		*63 (87.5)*
Indinavir	*5 (6.9)*	*4 (5.6)*	*2 (2.8)*	*1 (1.4)*	*60 (83.3)*
Saquinavir	*5 (6.9)*	*4 (5.6)*	*2 (2.8)*		*61 (84.7)*
Tipranavir	*1 (1.4)*	*3 (4.2)*	*1 (1.4)*	*2 (2.8)*	*65 (90.2)*
**NRTI (n = 72)**					
Tenofovir	3 (4.2)	9 (12.5)	9 (12.5)	2 (2.8)	49 (68.1)
Lamivudine	29 (40.3)		1 (1.4)		42 (58.3)
Abacavir	15 (20.8)	9 (12.5)	7 (9.7)		41 (56.9)
Didanosine	*17 (23.6)*	*10 (13.9)*	*2 (2.8)*	*7 (9.7)*	*36 (50)*
Stavudine	*14 (19.4)*	*8 (11.1)*	*8 (11.1)*	*1 (1.4)*	*41 (56.9)*
Zidovudine	15 (20.8)	4 (5.6)	4 (5.6)	1 (1.4)	48 (66.7)
**NNRTI (n = 72)**					
Efavirenz	34 (47.2)	8 (11.1)	1 (1.4)	8 (11.1)	34 (47.2)
Nevirapine	43 (59.7)	1 (1.4)		1 (1.4)	27 (37.5)
Doravirine	*6 (8.3)*	*12 (16.7)*	*7 (9.7)*	*5 (6.9)*	*42 (58.3)*
Etravirine	10 (13.9)	11 (15.3)	5 (6.9)	12 (16.7)	34 (47.2)
Rilpivirine	18 (25)	4 (5.6)	11 (15.3)	1 (1.4)	33 (45.8)
**INSTI (n = 84)**					
Dolutegravir	12 (14.3)	7 (8.3)		3 (3.6)	62 (73.8)
Bictegravir	11 (13.1)	8 (9.5)		3 (3.6)	62 (73.8)
Cabotegravir	19 (22.6)		3 (3.6)		62 (73.8)
Elvitegravir	16 (19.0)	3 (3.6)	3 (3.6)	1 (1.2)	61 (72.6)
Raltegravir	16 (19.0)	1 (1.2)	5 (5.9)	1 (1.2)	61 (72.6)

INSTI, integrase strand transfer inhibitor; NNRTI, non-nucleoside reverse transcriptase inhibitor; NRTI, nucleoside reverse transcriptase inhibitor; PI, protease inhibitor.

Italics represent drugs that are commonly used in the standard of care regimens in Zimbabwe.

Supplementary Table S5 Drug-class HIV-1 resistance patterns observed in patients failing DTG-based ART in Zimbabwe.

Drug-class resistance patterns	HIV-1 genotypes n = 54 (%)	TDF/3TC/DTG n = 20 (%)	ABC/3TC/DTG n = 22 (%)	AZT/3TC/DTG n = 10 (%)	TAF/XTC/DTG n = 2 (%)
**No resistance**	13 (24.1)	8 (40)	5 (22.7)	0 (0)	0 (0)
**Single-class resistance**					
INSTI	1 (1.9)	1 (5)	0	0 (0)	0 (0)
NRTI	3 (5.5)	0 (0)	3 (13.6)	0 (0)	0 (0)
NNRTI	10 (18.5)	3 (15)	6 (27.3)	1 (10)	0 (0)
PI	1 (1.9)	1 (5)	0 (0)	0 (0)	0 (0)
**Dual-class resistance**					
NRTI & INSTI	1 (1.9)	0 (0)	1 (4.5)	0 (0)	0 (0)
NNRTI & INSTI	0 (0)	0 (0)	0 (0)	0 (0)	0 (0
PI & INSTI	0 (0)	0 (0)	0 (0)	0 (0)	0 (0)
PI & NRTI	0 (0)	0 (0)	0 (0)	0 (0)	0 (0)
PI & NNRTI	0 (0)	0 (0)	0 (0)	0 (0)	0 (0)
NRTI & NNRTI	10 (18.5)	2 (10)	3 (13.6)	4 (40)	1 (50)
**Triple-class resistance**					
NRTI, NNRTI & INSTI	8 (10.9)	3 (15)	2 (9.1)	3 (30)	0 (0)
PI, NNRTI & INSTI	0 (0)	0 (0)	0 (0)	0 (0)	0 (0)
PI, NRTI & INSTI	0 (0)	0 (10)	0 (0)	0 (0)	0 (0)
PI, NRTI & NNRTI	6 (11.1)	2 (10)	2 (9.1)	1 (10)	1 (50)
**Four-class resistance**					
PI, NRTI, NNRTI & INSTI	1 (1.9)	0 (0)	0 (0)	1 (10)	0 (0)

3TC, lamivudine; ABC, abacavir; ART, antiretroviral therapy; AZT, zidovudine; DTG, dolutegravir; HIVDR, HIV drug resistance; IQR, interquartile range; TAF, tenofovir alafenamide; TDF, tenofovir disoproxil fumarate; INSTI, integrase strand transfer inhibitor; NNRTI, non-nucleoside reverse transcriptase inhibitor; NRTI, nucleoside reverse transcriptase inhibitor; PI, protease inhibitor.
